# Rationale and design of a multicenter randomized controlled study to evaluate the preventive effect of ipragliflozin on carotid atherosclerosis: the PROTECT study

**DOI:** 10.1186/s12933-016-0449-7

**Published:** 2016-09-13

**Authors:** Atsushi Tanaka, Toyoaki Murohara, Isao Taguchi, Kazuo Eguchi, Makoto Suzuki, Masafumi Kitakaze, Yasunori Sato, Tomoko Ishizu, Yukihito Higashi, Hirotsugu Yamada, Mamoru Nanasato, Michio Shimabukuro, Hiroki Teragawa, Shinichiro Ueda, Satoshi Kodera, Munehide Matsuhisa, Toshiaki Kadokami, Kazuomi Kario, Yoshihiko Nishio, Teruo Inoue, Koji Maemura, Jun-ichi Oyama, Mitsuru Ohishi, Masataka Sata, Hirofumi Tomiyama, Koichi Node, On behalf of the PROTECT Study Investigators

**Affiliations:** 1 Department of Cardiovascular Medicine, Saga University, Saga, Japan; 2 Department of Cardiology, Nagoya University Graduate School of Medicine, Nagoya, Japan; 3 Department of Cardiology, Dokkyo Medical University Koshigaya Hospital, Koshigaya, Japan; 4 Division of Cardiovascular Medicine, Department of Medicine, Jichi Medical University, Shimotsuke, Japan; 5 Cardiology Department, Kameda Medical Center, Kamogawa, Japan; 6 Department of Clinical Medicine and Development, National Cerebral and Cardiovascular Center, Osaka, Japan; 7 Department of Global Clinical Research, Graduate School of Medicine, Chiba University, Chiba, Japan; 8 Department of Clinical Laboratory Medicine, Faculty of Medicine, University of Tsukuba, Tsukuba, Japan; 9 Department of Cardiovascular Regeneration and Medicine, Research Institute for Radiation Biology and Medicine, Hiroshima University, Hiroshima, Japan; 10 Department of Cardiovascular Medicine, Tokushima University Hospital, Tokushima, Japan; 11 Cardiovascular Center, Japanese Red Cross Nagoya Daini Hospital, Nagoya, Japan; 12 Department of Cardio-Diabetes Medicine, Institute of Biomedical Sciences, Tokushima University Graduate School, Kuramoto, Japan; 13 Department of Cardiovascular Medicine, JR Hiroshima Hospital, Hiroshima, Japan; 14 Department of Clinical Pharmacology and Therapeutics, University of the Ryukyus, Nishihara, Japan; 15 Department of Cardiology, Asahi General Hospital, Chiba, Japan; 16 Diabetes Therapeutics and Research Center, Tokushima University, Tokushima, Japan; 17 Department of Cardiovascular Medicine, Saiseikai Futsukaichi Hospital, Chikushino, Japan; 18 Department of Diabetes and Endocrine Medicine, Kagoshima University Graduate School of Medical and Dental Sciences, Kagoshima, Japan; 19 Department of Cardiovascular Medicine, Dokkyo Medical University, Mibu, Japan; 20 Department of Cardiovascular Medicine, Nagasaki University Graduate School of Biomedical Sciences, Nagasaki, Japan; 21 Department of Cardiovascular Medicine and Hypertension, Kagoshima University Graduate School of Medical and Dental Sciences, Kagoshima, Japan; 22 Department of Cardiovascular Medicine, Institute of Biomedical Sciences, Tokushima University Graduate School, Tokushima, Japan; 23 Department of Cardiology, Tokyo Medical University, Tokyo, Japan

**Keywords:** Atherosclerosis, Intima-media thickness (IMT), Ipragliflozin, SGLT2 inhibitor, Type 2 diabetes mellitus

## Abstract

**Background:**

Type 2 diabetes mellitus is associated strongly with an increased risk of micro- and macro-vascular complications, leading to impaired quality of life and shortened life expectancy. In addition to appropriate glycemic control, multi-factorial intervention for a wide range of risk factors, such as hypertension and dyslipidemia, is crucial for management of diabetes. A recent cardiovascular outcome trial in diabetes patients with higher cardiovascular risk demonstrated that a SGLT2 inhibitor markedly reduced mortality, but not macro-vascular events. However, to date there is no clinical evidence regarding the therapeutic effects of SGLT2 inhibitors on arteriosclerosis. The ongoing PROTECT trial was designed to assess whether the SGLT2 inhibitors, ipragliflozin, prevented progression of carotid intima-media thickness in Japanese patients with type 2 diabetes mellitus.

**Methods:**

A total of 480 participants with type 2 diabetes mellitus with a HbA1c between 6 and 10 % despite receiving diet/exercise therapy and/or standard anti-diabetic agents for at least 3 months, will be randomized systematically (1:1) into either ipragliflozin or control (continuation of conventional therapy) groups. After randomization, ipragliflozin (50–100 mg once daily) will be added on to the background therapy in participants assigned to the ipragliflozin group. The primary endpoint of the study is the change in mean intima-media thickness of the common carotid artery from baseline to 24 months. Images of carotid intima-media thickness will be analyzed at a central core laboratory in a blinded manner. The key secondary endpoints include the change from baseline in other parameters of carotid intima-media thickness, various metabolic parameters, and renal function. Other cardiovascular functional tests are also planned for several sub-studies.

**Discussion:**

The PROTECT study is the first to assess the preventive effect of ipragliflozin on progression of carotid atherosclerosis using carotid intima-media thickness as a surrogate marker. The study has potential to clarify the protective effects of ipragliflozin on atherosclerosis.

*Trial registration* Unique Trial Number, UMIN000018440 (https://upload.umin.ac.jp/cgi-open-bin/ctr_e/ctr_view.cgi?recptno=R000021348)

**Electronic supplementary material:**

The online version of this article (doi:10.1186/s12933-016-0449-7) contains supplementary material, which is available to authorized users.

## Background

Type 2 diabetes mellitus (T2DM) is characterized by prolonged systemic insulin resistance, resultant impaired insulin insufficiency, and life-threatening micro- and macro-vascular complications [1–4]. The risk of cardiovascular (CV) disease is already increased in the pre-diabetic state of impaired glucose tolerance (IGT) and is associated to a greater degree with impaired fasting and/or 2-h plasma glucose than with HbA1c levels [5–8]. Abnormal glycemic metabolism therefore has a central role in diabetic pathophysiology. However, whether glucose-lowering treatments reduce the risk of future CV events still remains controversial, despite the legacy-effect of long-term intensive glycemic intervention [9–11]. Given the multi-factorial nature of T2DM progression, early medical intervention using a comprehensive approach according to an individual’s medical background needs to be emphasized in the management of the disorders [12, 13]. However, relevant risk factors are often not controlled optimally, and no conventional anti-diabetic agents can easily achieve such therapeutic goals. Given the worldwide increase in the number of patients with the metabolic syndrome including obesity and diabetes [14, 15], early establishment of therapeutic strategies to prevent the subsequent occurrence of obesity/diabetes-related CV complications is urgently required.

Sodium glucose cotransporter 2 (SGLT2) inhibitors are novel glucose-lowering agents that increase urinary glucose excretion by modulating selective inhibition of SGLT2 in the proximal renal tubule [16]. SGLT2 inhibitors alleviate glucotoxicity in an insulin-independent manner and improve beta-cell dysfunction, and therefore may have indirect metabolic benefits [17]. Of the SGLT2 inhibitors, ipragliflozin was developed in Japan. There is evidence that ipragliflozin has the favorable metabolic effects, including improved glycemic control, and decreased blood pressure (BP), body weight (BW), and visceral adipose tissue, indicating a potential CV protective effect [18, 19]. Several mega-clinical trials designed to clarify the effects of SGLT2 inhibitors on clinical CV outcomes are now in progress [20]. Of these trials, EMPA-REG OUTCOME trial showed that empagliflozin markedly reduced the risk of CV mortality compared to placebo [21]. Although CV mortality and worsening of heart failure were both decreased dramatically, empagliflozin treatment failed to reduce macro-vascular events, such as non-fatal myocardial infarction and stroke. The clinical impact of SGLT2 inhibitors on CV benefits has therefore attracted considerable attention, although the mechanisms by which SGLT2 inhibitors exert these benefits beyond glucose-lowering are not fully understood. In particular, clinical evidence regarding the therapeutic effect of SGLT2 inhibitors on arteriosclerosis in patients with diabetes is still lacking. The effects on arteriosclerosis of other anti-diabetic agents, such as pioglitazone and dipeptidyl peptidase 4 (DPP-4) inhibitors, have been evaluated in randomized clinical trials using surrogate markers, such as carotid intima-media thickness (IMT) [22–25]. This method is well-established and has good reproducibility and reliability to reflect the clinical severity of systemic atherosclerosis, and is therefore useful for evaluating drug efficacy.

On the basis of this background, the PROTECT study was designed to evaluate the anti-atherosclerotic effect of ipragliflozin using IMT as a surrogate marker for the risk of CV events and also to clarify the mechanisms by which SGLT2 inhibitors may improve CV outcomes. This study may provide novel evidence regarding SGLT2 inhibitor-mediated pharmacological intervention on carotid atherosclerosis.

## Methods

### Study overview and design

The PROTECT study is an ongoing, multicenter, prospective, randomized, open-label, blinded-endpoint, parallel group, investigator-initiated clinical trial (phase IV). The study will test the hypothesis that compared to standard care alone, the addition of ipragliflozin to standard care in T2DM may suppress the progression of carotid atherosclerosis, accompanied by an improvement in glycemic and lipid metabolism and vascular function. After recruitment and randomization of the patients into groups with or without ipragliflozin, each treatment is continued for 24 months, and the long-term safety and effects of ipragliflozin on CV systems then evaluated.

The study protocol was approved by the local institutional review boards and independent ethics committees. The study will be conducted in full compliance with the articles of the Declaration of Helsinki and according to the Ethical Guidelines for Medical and Health Research Involving Human Subjects established by the Ministry of Health, Labour, and Welfare and Ministry of Education, Culture, Sports, Science, and Technology. The PROTECT study was registered by the UMIN in July 2015 (ID: 000018440).

### Study population and recruitment

We aim to recruit a total of 480 participants across approximately 35 sites in Japan. Recruitment for the study began in September 2015 and will end in December 2017. Eligible participants for the study are T2DM patients who comply with all the enrollment criteria. The detailed inclusion and exclusion criteria are listed in Table 1. Briefly, patients will be enrolled if they are aged ≥20 year, diagnosed as having T2DM in accordance with the Japanese guidelines [26], with a HbA1c between 6.0 and 10.0 % despite diet and exercise therapy and/or taking standard medications for at least 3 months prior to randomization. After initial screening using previous medical records, each participant is required to receive an adequate explanation of the study plan, with written informed consent then being obtained.

**Table 1:** Detailed inclusion and exclusion criteria

Inclusion	Exclusion
Adults (aged ≥20 years)	Type 1 diabetes mellitus
T2DM with 6.0 % ≤ HbA1c < 10.0 % despite diet and exercise therapy and/or the standard medications for at least 3 months prior to randomization	History of severe ketosis, diabetic coma, or
The patient provided written informed consent to participate in the study	Precoma attack ≤6 months prior to informed consent
	Patients with severe infection or trauma at trial screening
	Patients in perioperative period around trial screening
	Severe renal dysfunction (eGFR **<** 45 ml/min/1.73 m^2^) or patients receiving dialysis
	History of coronary artery disease, coronary vascularization, open-heart surgery, stroke, or transient ischemic attack ≤3 months prior to eligibility
	CHF (NYHA functional classification III and IV)
	History of administration of SGLT2 inhibitor 1 month prior to study initiation
	Pregnant or suspected pregnancy in females
	Lactating female
	History of hypersensitivity to ingredients of ipragliflozin
	Considered inappropriate for the study by investigators due to other reasons, such as malignancy

*CHF* chronic heart failure, *eGFR* estimated glomerular filtration rate, *NYHA* New York Heart Association, *SGLT2* sodium glucose cotransporter 2, *T2DM* type 2 diabetes mellitus

### Study outline and follow up

**Fig. 1:**
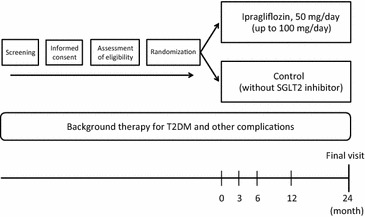
Study outline

After informed consent has been obtained and the eligibility assessment is completed, all eligible participants will be randomized and assigned into either the ipragliflozin group or standard-care (control) group. Follow-up visits are scheduled at 3, 6, 12 and 24 months (Fig. 1). All participants will see their usual-care physicians at each visit to receive usual-care and individualized appropriate treatment according to their background disease, in addition to administration of the study drug.

### Randomization and treatment

Eligible participants with appropriately signed informed consent will be randomized to either the ipragliflozin group or control group (ratio 1:1) using the web-based minimization method with biased coin assignment balancing [27, 28] for age (<65, ≥65 year), HbA1c level (<7.0, ≥7.0 %), systolic BP (<135, ≥135 mmHg), use of statins, and use of biguanides at the time of screening.

All participants will be followed-up for 24 months. Although a specific numerical goal in glycemic control such as HbA1c level is not set for the study, all participants need to be treated to achieve a personalized goal recommended by the treatment guideline in Japan (details in Additional file 1) [26]. Participants who are assigned to the ipragliflozin group receive ipragliflozin 50 mg once daily in addition to their background medical therapy. In accordance with official recommendation regarding use of SGLT2 inhibitor from the Japan Diabetes Society [29], patients aged ≥75 years should be most carefully followed up with particular attention to development of volume depletion-related adverse drug reactions [30]. If the personalized goal is not achieved, the dose of ipragliflozin can be increased by the investigators to 100 mg once daily. Participants who are assigned to the control group continue their background therapy. Within the appropriate range of the therapeutic goal, the participant’s background therapy will be, in principle and if possible, unchanged during the study in both groups. However, if participants cannot achieve their glycemic goal, co-administration of anti-diabetic agents other than SGLT2 inhibitors or increased dosages of the other anti-diabetic agents in both groups may be considered by investigators, with caution being taken to prevent the development of hypoglycemia. However, because pioglitazone is known to have a suppressive effect on the progression of IMT, compared to glimepiride [22], it is prohibited to prescribe it or change its dose during the study. After the study is completed, all participants can continue any anti-diabetic treatment in accordance with their individual condition.

### Measurements

Baseline characteristics, including gender, age, body height and weight, abdominal circumference, complications, duration of T2DM, background treatment, and smoking and drinking habits will be recorded prior to randomization. The status of the study medications and the participant’s background treatment will be recorded at each visit. Measurements of BP, pulse rate, BW, and body mass index (BMI) will also be carried out at baseline and after 12 and 24 months. Abdominal circumference will be measured at baseline and 24 months. Blood tests without HbA1c level will be checked at baseline and 24 months (details listed in Additional file 2); HbA1c will be measured at baseline and after 12 and 24 months. Specific biomarkers such as N-terminal pro-brain natriuretic peptide (NT-proBNP), high-sensitivity C reactive protein (hsCRP), high-molecular weight adiponectin, and malondialdehyde modified low-density lipoprotein (MDA-LDL) will be measured at baseline and 24 months. Creatinine-corrected urinary albumin excretion will be measured at baseline and 24 months (optional). Some optional imaging and physiological tests are also planned in the study including abdominal computed tomography to measure the amount of visceral and subcutaneous fat, echocardiograms, flow-mediated dilation (FMD), pulse-wave velocity (PWV), cardio-ankle vascular index (CAVI), and augmentation index (AI) (details listed in Additional file 3).

#### Measurement of carotid IMT

**Fig. 2:**
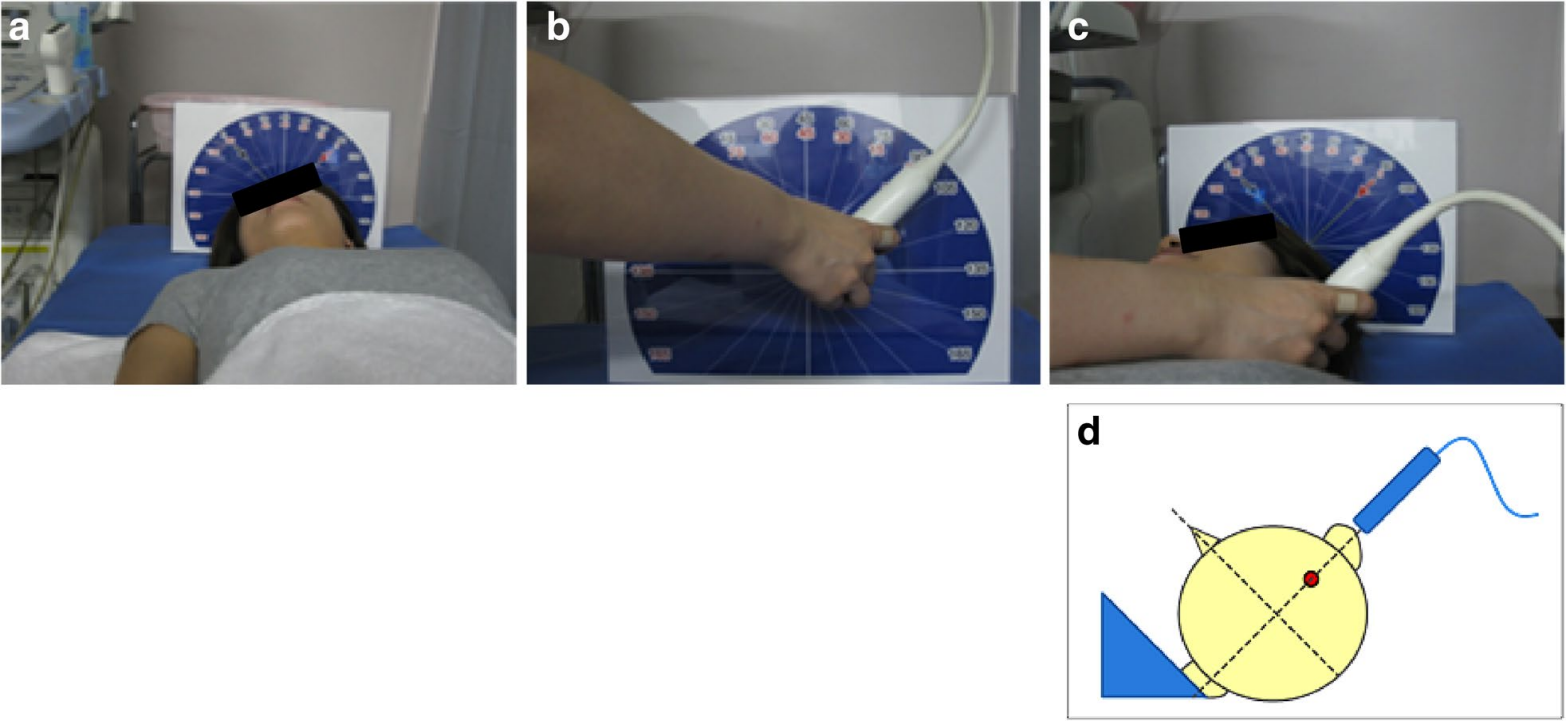
Method for measuring IMT. **a** Head position is set at 45° toward the other side (*right*) when measuring at the left carotid artery. **b** The probe angle is also set at 45° using the ruler on the test side. **c** A plus B. **d** Schema for measuring the left carotid artery. The probe is set perpendicular to the sagittal plane

The protocol and method for measuring carotid IMT have been described in detail previously [25, 31, 32]. In brief, the carotid ultrasound examinations using standardized imaging protocols and systems equipped with >7.5 MHz linear transducers will be performed at each local site and then measured at a core laboratory (Tsukuba University) at baseline and 24 months after randomization. Expert trained sonographers who have attended a lecture on measuring carotid IMT will carry out the procedure according to the Mannheim carotid IMT consensus [33, 34]. The head position and probe angle of the ultrasound approach will be set using a ruler located just cephalad (Fig. 2). Longitudinal B-mode images, perpendicular to the ultrasound beam, with a 3–4 cm imaging depth, will be recorded in the distal common carotid arteries (CCA), carotid bulbs, and proximal internal carotid arteries (ICA) on both sides. The lateral probe incidence is used to obtain CCA images, using external landmarks and an original semicircular protractor developed for this purpose. The mean CCA-IMT indicates the average IMT value of the right and left CCA-IMT, 10 mm from the bulb. The following far wall IMTs will be measured; maximum IMT of the CCA and mean and maximum IMTs of the bulb and ICA. The optimized R-wave gated still frames of the carotid IMT will be stored as JPEG files, with all the parameters collected and measured at the core laboratory. An expert analyzer blinded to the allocation and clinical information of the subjects will measure all the IMT values using an automatic IMT measurement software program (Vascular Research Tools 5, Medical Imaging Applications, Iowa, USA) [35]. The software program identifies the lumen/intima and the media/adventitia borders in this region and calculates the distance between them.

### Safety

Based on the intention-to-treat the entire population, safety will be checked by recording the following adverse effects (AEs) throughout the study: severe AEs regardless as to whether or not there is causal relationship between the AEs and the study; and relevant AEs such as hypoglycemia, genital or urinary tract infections, ketoacidosis, and hypovolemic symptoms. When the investigators confirm these AEs, the grade of severity, procedures, outcomes, and relationship to the study drug will be assessed. A prompt report to the study secretariat and to the Data and Safety Monitoring Board (DSMB) will then be made by the trial organizer. The members of the DSMB consist of authorized endocrinologists, cardiologists, or neurologist with relevant expertise. The criteria for withdrawal from the trial are listed in Table 2. The incident of withdrawal from the study will be reported promptly to the DSMB by the chief investigator. The DSMB will then deliberate on the incident and report the decision to the chief investigator.

**Table 2:** Discontinuance criteria

Severe hypoglycemia
Seriously poor glycemic control such as ≥HbA1c 12.0 % confirmed by second measurement on different day
Offer for participation declined by participants
Deviancy of eligibility after registration
Considered inappropriate to continue the study by investigators due to aggravation of primary disease or complications
Considered inappropriate to continue the study by investigators due to adverse side effects of the study drug
Pregnant
Poor drug adherence (<75 %, or >120 %)
Considered inappropriate to continue the study by investigators due to some other reason

### Study endpoints

Intima-media thickness (IMT) in the carotid artery is well-established as a surrogate marker for risk of CV diseases and is also useful for evaluating the effectiveness of various types of therapeutic interventions in patients with or without T2DM [35–37]. The primary endpoint in the study is the change in mean IMT of the CCA from baseline to 24 months. The secondary endpoints are the values and changes in parameters after 24 months of treatment, including: (1) mean IMT of the bulb and ICA, (2) max IMT of the CCA, bulb, and ICA, (3) the overall mean of mean IMT of the CCA, bulb, and ICA, (4) the mean of max IMT of the CCA, bulb, and ICA, (5) specific biomarkers including hsCRP, MDA-LDL, NT-proBNP, and high-molecular weight adiponectin, (6) the cardiovascular functional tests listed in Additional file 3 (optional), (7) abdominal circumference and amount of visceral and subcutaneous fat measured by abdominal computed tomography (optional), (8) creatinine-corrected urinary albumin excretion. In addition, the values and changes after 12 and 24 months in several clinical parameters including BP, BW, and BMI and laboratory data (details listed in Additional file 2) will be evaluated. Safety endpoints also include AEs and adverse drug reactions observed during the study.

### Statistical considerations

#### Sample size estimation

Due to the lack of data on the effect of SGLT2 inhibitors on carotid IMT, we referred to the statistical data from the CHICAGO study [22] and PROLOGUE study [25]. In the CHICAGO study, pioglitazone caused a significant inhibition of the progression of CCA-IMT (−0.0010 mm after 72 weeks), compared to glimepiride (+0.0120 mm after 72 weeks). We estimated the changes from baseline would be −0.0013 mm (pioglitazone) and +0.016 mm (glimepiride) after 96 weeks. Based on the assumption that ipragliflozin may inhibit the progression of CCA-IMT to the same extent as pioglitazone, we estimated the group difference as 0.016 (ipragliflozin −0.001 and control +0.015) ±0.06 (standard deviation). At a significant level of 5 % (two-sided), the sample size of 222 patients per arm provides a power of 80 % for each comparison. Allowing for a dropout rate of 5 %, 240 patients in each arm (a total of 480 patients) provides sufficient statistical power for the study.

#### Statistical analysis plan

The analyses of the primary and secondary endpoints will be performed in the full analysis set (FAS), which includes all participants who received at least one dose of treatment during the study period and did not have any serious violation of the study protocol such as not providing informed consent, registration outside of the study period, or data collected after commencement of treatment.

Summary statistics will be calculated for the baseline characteristics including the frequencies and proportions for categorical variables and means ± standard deviations for continuous variables. The patient characteristics will be compared using Chi square tests for categorical variables, t tests for normally distributed continuous variables, or the Wilcoxon rank sum tests for continuous variables with a skewed distribution.

The analysis plan is similar to that used in previous studies we have conducted [25, 31, 32]. In brief, for the primary analysis comparing treatment effects, the baseline-adjusted means and their 95 % confidence intervals, estimated by analysis of covariance of the change in average carotid IMT at 24 months, will be compared between the treatment groups (ipragliflozin group vs. control group). The results will be adjusted by allocation factors. The primary analysis will not include missing observations, with the mixed effects model for repeated measures (MMRM) being used as a sensitivity analysis to examine the effect of missing data. In addition, MMRM will be used as a sensitivity analysis to examine the outcomes at baseline and 24 months modelled as a function of time, treatment, and treatment-by-time interaction. The secondary analysis will be performed in the same manner as the primary analysis.

All comparisons are planned and all *P* values will be two sided. *P* values <0.05 will be considered statistically significant. All statistical analyses will be performed using SAS software version 9.4 (SAS Institute, Cary, NC, USA). The statistical analysis plan will be developed by the principal investigator and a biostatistician before completion of patient recruitment and database lock.

### Study organization and oversight

The principal investigators of the PROTECT study are (details in Additional file 4) Koichi Node (Chief), Department of Cardiovascular Medicine, Saga University and Toyoaki Murohara, Department of Cardiology, Nagoya University Graduate School of Medicine. The research advisor is Masafumi Kitakaze, Department of Clinical Medicine and Development, National Cerebral and Cardiovascular Center. The steering committee will carry out planning, operating, analyzing, and presentation of the trial. The executive committee will supervise the trial design and operation of the study. The roles of the DSMB are described in the section on Safety. The trial secretariat is in DOT INTERNATIONAL CO., LTD, Tokyo, Japan. Each data management, monitoring, statistical analyses, and audit will be implemented independently on the basis of the outsourcing agreement. Carotid IMT will be measured at a core laboratory, Tsukuba University. Data monitoring will be enforced to ensure the research is performed properly, with an independent audit team inspecting several main institutes to ensure the quality of the study data.

## Discussion

The PROTECT study is an ongoing, multicenter, prospective, randomized, investigator-initiated clinical trial that has the aim of assessing the add-on effect of ipragliflozin using carotid IMT as a surrogate marker of CV risk. Cardiac and vascular functional tests will also be evaluated as secondary endpoints. Eligible patients with T2DM will be assigned to ipragliflozin or conventional standard care groups. The primary endpoint is the change in mean IMT of the CCA from baseline to 24 months of treatment. The study has the potential to provide novel clinical evidence on the anti-atherosclerotic effect of ipragliflozin.

Carotid IMT is used widely as a noninvasive measure of systemic atherosclerotic state and to predict subsequent CV events and mortality [38, 39]. A number of studies have demonstrated that increased IMT correlates strongly with the risk of future CV disease in a wide range of populations, especially T2DM patients [40–44]. Measuring carotid IMT is also recognized as a useful surrogate marker for evaluating the efficacy of therapeutic interventions on CV risk factors and atherosclerotic diseases [37, 45–47]. Although the current study is a multicenter open-label design, repeated IMT measurements are planned in a blinded manner. The analyses will be carried out at a core laboratory according to global recommendations in order to avoid bias and measurement error between institutions [48]. The same systematic procedures for analysis of carotid IMT were used in our previous and other ongoing studies [25, 31, 32]. The reliability and reproducibility of measurements of carotid IMT will be highly certified in the current study.

Because diabetes contributes strongly to accelerated progression of carotid IMT [49], the inhibitory effects of several anti-diabetic agents on carotid IMT progression have been investigated extensively. In the CHICAGO study [22], mean and max carotid IMT progression was significantly lower in the pioglitazone group compared to the glimepiride group. This inhibitory effect has been confirmed in other clinical trials [50] and is, in part, consistent with the result from a large-scale outcome study, the Prospective Pioglitazone Clinical Trial in Macrovascular Events (PROactive). That study demonstrated that the addition of pioglitazone was associated with a 16 % risk reduction in the composite of all-cause mortality and non-fatal macro-vascular events compared to the addition of placebo [51]. Even in the IGT subjects, acarbose, an alpha-glucosidase inhibitor, also attenuated significantly the mean IMT progression relative to placebo [52]. This result may provide a possible mechanism by which acarbose reduced the incidence of cardiovascular events in the earlier trial [53]. Regarding DPP-4 inhibitors, the TECOS trial that evaluated CV outcomes in 14,671 T2DM patients with established CV disease showed a neutral effect of sitagliptin on the risk of major adverse CV events during a median follow-up of 3 years [54]. The other outcome mega-trials that evaluated CV safety of DPP-4 inhibitors, the EXAMINE and SAVOR-TIMI 53 studies, also showed similar results [55, 56]. Interestingly, in the Program of Vascular Evaluation under Glucose Control by a DPP-4 Inhibitor (PROLOGUE), sitagliptin failed to inhibit the progression of carotid IMT compared to standard diabetes care during 24 months of follow-up [25]. In contrast, other studies of DPP-4 inhibitors have demonstrated a beneficial effect on progression of carotid IMT [23, 24]. The reasons for this discrepancy remain uncertain, although clinical differences in the patients’ background, such as concomitant drugs and severity of diabetes and CV risk may, in part, influence the effectiveness of DPP-4 inhibitors on carotid atherosclerosis. Recent clinical trials also clearly show a close association between anti-diabetic agents-mediated changes in carotid IMT and CV outcomes in the majority of T2DM patients.

SGLT2 inhibitors are a novel class of oral anti-diabetic agent that lower blood glucose level by increasing urinary glucose excretion. In addition to the glycemic pathway, SGLT2 inhibitors are associated closely with non-glycemic modifications, such as hemodynamic, metabolic, renal, and neurohormonal effects [20, 57]. In 2015, the EMPA-REG OUTCOME trial reported outstanding results that the SGLT2 inhibitor, empagliflozin, markedly improved clinical outcomes in diabetes patients with a higher CV risk [21]. Because other outcome trials using SGLT2 inhibitors other than empagliflozin are now ongoing [58–60], it still remains to be determined whether this clinical impact is a class effect of SGLT2 inhibitors. However, given their mode of action and favorable effects on the entire CV system, it is very likely that further positive evidence may be obtained [61]. In the EMPA-REG OUTCOME trial, empagliflozin caused a significant reduction in CV mortality and hospitalization for worsened heart failure rather than macrovascular complications, such as non-fatal myocardial infarction and stroke. Based on these beneficial clinical outcomes, possible mechanisms may be largely hemodynamic effects induced by glycosuria and natriuresis, rather than a direct anti-atherothrombotic effect [62–64]. However, SGLT2 inhibitors ameliorate various risk factors related to CV disease, such as BP, BW, uric acid, and lipid profiles independent of glycemic control per sé, suggesting the possible existence of anti-atherosclerotic actions. Indeed, there is evidence that SGLT2 inhibitors prevent excess oxidative stress and inflammation in animal models [65–69]. In clinical studies, direct effects on arterial stiffness were also observed in patients with type 1and type 2 diabetes [70, 71]. Although the increased incidence of non-fatal stroke was reported in the EMPA-REG OUTCOME trial and subsequent meta-analyses [21, 61], the study duration may have been too short to prevent the occurrence of atherogenic macro-vascular events, including stroke. Importantly, the precise effects of SGLT2 inhibitor on local and systemic atherosclerosis in clinical settings have proved elusive. It would therefore be plausible to implement a mechanistic study using a surrogate marker as a study endpoint.

In the current study, we are attempting to assess the preventive effect of a SGLT2 inhibitor, ipragliflozin, on carotid IMT progression. In 2014, ipragliflozin was the first SGLT2 inhibitor to be released in Japan [19]. Tahara et al. [72] reported that compared to other SGLT2 inhibitors, ipragliflozin had a relatively longer-acting and earlier-onset of action on renal SGLT2. Accumulated evidence from the initial clinical studies in Japanese T2DM patients also showed short- and long-term favorable effects of ipragliflozin on glycemic, metabolic, and safety parameters [30, 73–77]. Takahara et al. [78] also reported that ipragliflozin treatment improved pancreatic beta-cell dysfunction and subsequent insulin resistance in T2DM patients, similar to that reported for other SGLT2 inhibitors [79, 80]. Systemic insulin resistance (IR) plays a pivotal role in the pathogenesis and progression of obesity and noninsulin-dependent diabetes mellitus [81]. It is also known that insulin resistance and resultant diabetes are associated closely with non-alcoholic fatty liver disease (NAFLD), including non-alcoholic steatohepatitis (NASH); a progressive phenotype in the NAFLD spectrum [82–84]. Recent animal studies showed that ipragliflozin treatment attenuated liver dysfunction mediated by steatosis and fibrosis in some rodent models of NASH [85, 86]. Because SGLT2 is not expressed in the liver, such treatment effects may be caused indirectly by amelioration of systemic IR and inflammation. Treatment with a SGLT2 inhibitor therefore has the potential to improve obesity and diabetes-associated metabolic abnormalities in the entire body, suggesting the possibility of an anti-atherosclerotic action.

This study has several limitations. First, the PROTECT study is not a double blind placebo-controlled trial, but rather an open label design. Unexpected bias towards the assessment of outcomes resulting from the physicians’ choice of treatment may occur. To avoid this possible bias, there are strict requirements that the participants’ background treatment will, in principle and if possible, remain unchanged during the study. In addition, carotid IMT, a key endpoint in the study, will be measured at a central laboratory, and all the data will be managed and statistically analyzed in a blinded fashion. Second, because the duration of the study is 24 months, it is possible the additional anti-diabetic agents administered when glycemic control becomes worse, especially in the control group, may influence outcomes. It is therefore important to take into account that pioglitazone and some other DPP-4 inhibitors may also prevent progression of carotid IMT in patients with T2DM [22–24]. Third, because the investigators who are participating in the PROTECT study are mainly cardiologists, there may be potential variety of treatment or judgement in the management of diabetes. Therefore, the Steering Committee recommends clinical practice will be performed according to the participants’ comprehensive conditions based on the treatment guideline in Japan [26]. Last, patient’s renal function, estimated glomerular filtration rate (eGFR), is not included as an allocation factor, although patients with severe renal dysfunction (eGFR < 45 ml/min/1.73 m^2^) or receiving dialysis are excluded. The patient’s kidney function is one of major determinants of urinary glucose excretion by SGLT2 inhibitor treatment. Previous studies reported that urinary glucose excretion in patients with lower levels of eGFR was actually decreased, and improvement of glycemic control was lower than patients without impairment of renal function [87, 88]. However, there were SGLT2 inhibitor-induced reductions in body weight and blood pressure independently of patient’s renal function [89]. Thus, we have speculated that anti-atherosclerotic effect may be, in part, caused by ipragliflozin independently of glycemic control and renal function at baseline.

In summary, the PROTECT study is the first to evaluate the effect of ipragliflozin on carotid IMT in patients with T2DM. Clear evidence of the therapeutic effects of SGLT2 inhibitors on atherosclerosis is currently lacking in clinical settings. Given the multi-factorial effects of SGLT2 inhibitors independent of glycemic control, it is not unexpected that ipragliflozin is able to exert a protective action against the atherosclerotic process. This study has the potential to provide new knowledge on effective treatment to prevent atherogenic complications in patients with T2DM.

## Additional files

10.1186/s12933-016-0449-7 Guidepost for appropriate glycemic control in Japan HbA1c < 6.0 %.
10.1186/s12933-016-0449-7 Blood examination.
10.1186/s12933-016-0449-7 Cardiovascular functional tests.
10.1186/s12933-016-0449-7 Trial organization.

## Abbreviations

AEs: adverse effects
AI: augmentation index
BMI: body mass index
BP: blood pressure
BW: body weight
CAVI: cardio-ankle vascular index
CCA: common carotid artery
CV: cardiovascular
DPP-4: dipeptidyl peptidase 4
DSMB: data and safety monitoring board
eGFR: estimated glomerular filtration rate
FAS: full analysis set
FMD: flow-mediated dilation
hsCRP: high-sensitivity C reactive protein
ICA: internal carotid artery
IGT: impaired glucose tolerance
IMT: intima-media thickness
IR: insulin resistance
MDA-LDL: malondialdehyde modified low-density lipoprotein
MMRM: mixed effects model for repeated measures
NAFLD: non-alcoholic fatty liver disease
NASH: non-alcoholic steatohepatitis
NT-proBNP: N-terminal pro-brain natriuretic peptide
PWV: pulse-wave velocity
SGLT2: sodium glucose cotransporter 2
T2DM: type 2 diabetes mellitus

## References

[1] Defronzo RA (2009). Banting lecture. From the triumvirate to the ominous octet: a new paradigm for the treatment of type 2 diabetes mellitus. Diabetes.

[2] Nolan CJ, Damm P, Prentki M (2011). Type 2 diabetes across generations: from pathophysiology to prevention and management. Lancet.

[3] Bethel MA, Sloan FA, Belsky D, Feinglos MN (2007). Longitudinal incidence and prevalence of adverse outcomes of diabetes mellitus in elderly patients. Arch Intern Med.

[4] Kuusisto J, Laakso M (2013). Update on type 2 diabetes as a cardiovascular disease risk equivalent. Curr Cardiol Rep.

[5] Nakagami T (2004). Hyperglycaemia and mortality from all causes and from cardiovascular disease in five populations of Asian origin. Diabetologia.

[6] Sorkin JD, Muller DC, Fleg JL, Andres R (2005). The relation of fasting and 2-h postchallenge plasma glucose concentrations to mortality: data from the Baltimore Longitudinal Study of Aging with a critical review of the literature. Diabetes Care.

[7] Barr EL, Zimmet PZ, Welborn TA, Jolley D, Magliano DJ, Dunstan DW, Cameron AJ, Dwyer T, Taylor HR, Tonkin AM (2007). Risk of cardiovascular and all-cause mortality in individuals with diabetes mellitus, impaired fasting glucose, and impaired glucose tolerance: the Australian Diabetes, Obesity, and Lifestyle Study (AusDiab). Circulation.

[8] Barr EL, Boyko EJ, Zimmet PZ, Wolfe R, Tonkin AM, Shaw JE (2009). Continuous relationships between non-diabetic hyperglycaemia and both cardiovascular disease and all-cause mortality: the Australian Diabetes, Obesity, and Lifestyle (AusDiab) study. Diabetologia.

[9] Skyler JS, Bergenstal R, Bonow RO, Buse J, Deedwania P, Gale EA, Howard BV, Kirkman MS, Kosiborod M, Reaven P (2009). Intensive glycemic control and the prevention of cardiovascular events: implications of the ACCORD, ADVANCE, and VA diabetes trials: a position statement of the American Diabetes Association and a scientific statement of the American College of Cardiology Foundation and the American Heart Association. Diabetes Care.

[10] Zarich SW (2009). Antidiabetic agents and cardiovascular risk in type 2 diabetes. Nat Rev Endocrinol.

[11] Holman RR, Paul SK, Bethel MA, Matthews DR, Neil HA (2008). 10-year follow-up of intensive glucose control in type 2 diabetes. N Engl J Med.

[12] Dailey G (2011). Early and intensive therapy for management of hyperglycemia and cardiovascular risk factors in patients with type 2 diabetes. Clin Ther.

[13] Gaede P, Lund-Andersen H, Parving HH, Pedersen O (2008). Effect of a multifactorial intervention on mortality in type 2 diabetes. N Engl J Med.

[14] NCD Risk Factor Collaboration (2016). (NCD-RisC): trends in adult body-mass index in 200 countries from 1975 to 2014: a pooled analysis of 1698 population-based measurement studies with 19.2 million participants. Lancet.

[15] Danaei G, Finucane MM, Lu Y, Singh GM, Cowan MJ, Paciorek CJ, Lin JK, Farzadfar F, Khang YH, Stevens GA (2011). National, regional, and global trends in fasting plasma glucose and diabetes prevalence since 1980: systematic analysis of health examination surveys and epidemiological studies with 370 country-years and 2.7 million participants. Lancet.

[16] Abdul-Ghani MA, Norton L, Defronzo RA (2011). Role of sodium–glucose cotransporter 2 (SGLT 2) inhibitors in the treatment of type 2 diabetes. Endocr Rev.

[17] Scheen AJ, Paquot N (2014). Metabolic effects of SGLT-2 inhibitors beyond increased glucosuria: a review of the clinical evidence. Diabetes Metab.

[18] Ohkura T (2015). Ipragliflozin: a novel sodium–glucose cotransporter 2 inhibitor developed in Japan. World J Diabetes.

[19] Kurosaki E, Ogasawara H (2013). Ipragliflozin and other sodium–glucose cotransporter-2 (SGLT2) inhibitors in the treatment of type 2 diabetes: preclinical and clinical data. Pharmacol Ther.

[20] Inzucchi SE, Zinman B, Wanner C, Ferrari R, Fitchett D, Hantel S, Espadero RM, Woerle HJ, Broedl UC, Johansen OE (2015). SGLT-2 inhibitors and cardiovascular risk: proposed pathways and review of ongoing outcome trials. Diabetes Vasc Dis Res.

[21] Zinman B, Wanner C, Lachin JM, Fitchett D, Bluhmki E, Hantel S, Mattheus M, Devins T, Johansen OE, Woerle HJ (2015). Empagliflozin, cardiovascular outcomes, and mortality in type 2 diabetes. N Engl J Med.

[22] Mazzone T, Meyer PM, Feinstein SB, Davidson MH, Kondos GT, D’Agostino RB, Perez A, Provost JC, Haffner SM (2006). Effect of pioglitazone compared with glimepiride on carotid intima-media thickness in type 2 diabetes: a randomized trial. JAMA.

[23] Mita T, Katakami N, Shiraiwa T, Yoshii H, Onuma T, Kuribayashi N, Osonoi T, Kaneto H, Kosugi K, Umayahara Y (2016). Sitagliptin attenuates the progression of carotid intima-media thickening in insulin-treated patients with type 2 diabetes: the sitagliptin preventive study of intima-media thickness evaluation (SPIKE): a randomized controlled trial. Diabetes Care.

[24] Mita T, Katakami N, Yoshii H, Onuma T, Kaneto H, Osonoi T, Shiraiwa T, Kosugi K, Umayahara Y, Yamamoto T (2016). Alogliptin, a dipeptidyl peptidase 4 inhibitor, prevents the progression of carotid atherosclerosis in patients with type 2 diabetes: the study of preventive effects of alogliptin on diabetic atherosclerosis (SPEAD-A). Diabetes Care.

[25] Oyama J, Murohara T, Kitakaze M, Ishizu T, Sato Y, Kitagawa K, Kamiya H, Ajioka M, Ishihara M, Dai K (2016). The effect of sitagliptin on carotid artery atherosclerosis in patients with type 2 diabetes: the PROLOGUE randomized controlled trial. PLoS Med.

[26] The Japan Diabetes Society: treatment guide for diabetes 2014–2015. 2014, BUNKODO. 2014.

[27] Taves DR (1974). Minimization: a new method of assigning patients to treatment and control groups. Clin Pharmacol Ther.

[28] Pocock SJ, Simon R (1975). Sequential treatment assignment with balancing for prognostic factors in the controlled clinical trial. Biometrics.

[29] The Japan Diabetes Society: Recommendation for appropriate use of SGLT2 inhibitor. 2016. (in Japanese). http://www.fa.kyorin.co.jp/jds/uploads/recommendation_SGLT2.pdf Accessed 15 Aug 2016.

[30] Terauchi Y, Yokote K, Nakamura I, Sugamori H (2016). Safety of ipragliflozin in elderly Japanese patients with type 2 diabetes mellitus (STELLA-ELDER): interim results of a post-marketing surveillance study. Expert Opin Pharmacother.

[31] Oyama J, Ishizu T, Sato Y, Kodama K, Bando YK, Murohara T, Node K (2014). Rationale and design of a study to evaluate the effects of sitagliptin on atherosclerosis in patients with diabetes mellitus: PROLOGUE study. Int J Cardiol.

[32] Oyama J, Tanaka A, Sato Y, Tomiyama H, Sata M, Ishizu T, Taguchi I, Kuroyanagi T, Teragawa H, Ishizaka N (2016). Rationale and design of a multicenter randomized study for evaluating vascular function under uric acid control using the xanthine oxidase inhibitor, febuxostat: the PRIZE study. Cardiovasc Diabetol.

[33] Stein JH, Korcarz CE, Hurst RT, Lonn E, Kendall CB, Mohler ER, Najjar SS, Rembold CM, Post WS (2008). Use of carotid ultrasound to identify subclinical vascular disease and evaluate cardiovascular disease risk: a consensus statement from the American Society of Echocardiography Carotid Intima-Media Thickness Task Force. Endorsed by the Society for Vascular Medicine. J Am Soc Echocardiogr.

[34] Touboul PJ, Hennerici MG, Meairs S, Adams H, Amarenco P, Desvarieux M, Ebrahim S, Fatar M, Hernandez Hernandez R, Kownator S (2004). Mannheim intima-media thickness consensus. Cerebrovasc Dis.

[35] Lundby-Christensen L, Almdal TP, Carstensen B, Tarnow L, Wiinberg N (2010). Carotid intima-media thickness in individuals with and without type 2 diabetes: a reproducibility study. Cardiovasc Diabetol.

[36] Yokoyama H, Katakami N, Yamasaki Y (2006). Recent advances of intervention to inhibit progression of carotid intima-media thickness in patients with type 2 diabetes mellitus. Stroke.

[37] Sibal L, Agarwal SC, Home PD (2011). Carotid intima-media thickness as a surrogate marker of cardiovascular disease in diabetes. Diabetes Metab Syndr Obes.

[38] Simon A, Megnien JL, Chironi G (2010). The value of carotid intima-media thickness for predicting cardiovascular risk. Arterioscler Thromb Vasc Biol.

[39] Cao JJ, Arnold AM, Manolio TA, Polak JF, Psaty BM, Hirsch CH, Kuller LH, Cushman M (2007). Association of carotid artery intima-media thickness, plaques, and C-reactive protein with future cardiovascular disease and all-cause mortality: the Cardiovascular Health Study. Circulation.

[40] Lorenz MW, Markus HS, Bots ML, Rosvall M, Sitzer M (2007). Prediction of clinical cardiovascular events with carotid intima-media thickness: a systematic review and meta-analysis. Circulation.

[41] Lorenz MW, von Kegler S, Steinmetz H, Markus HS, Sitzer M (2006). Carotid intima-media thickening indicates a higher vascular risk across a wide age range: prospective data from the Carotid Atherosclerosis Progression Study (CAPS). Stroke.

[42] Bernard S, Serusclat A, Targe F, Charriere S, Roth O, Beaune J, Berthezene F, Moulin P (2005). Incremental predictive value of carotid ultrasonography in the assessment of coronary risk in a cohort of asymptomatic type 2 diabetic subjects. Diabetes Care.

[43] Mitsuhashi N, Onuma T, Kubo S, Takayanagi N, Honda M, Kawamori R (2002). Coronary artery disease and carotid artery intima-media thickness in Japanese type 2 diabetic patients. Diabetes Care.

[44] Kasami R, Kaneto H, Katakami N, Sumitsuji S, Yamasaki K, Kuroda T, Tachibana K, Yasuda T, Kuroda A, Matsuoka TA (2011). Relationship between carotid intima-media thickness and the presence and extent of coronary stenosis in type 2 diabetic patients with carotid atherosclerosis but without history of coronary artery disease. Diabetes Care.

[45] Csiba L (2005). Carotid intima-media thickness measured by ultrasonography: effect of different pharmacotherapies on atherosclerosis progression. Orv Hetil.

[46] Bots ML, Evans GW, Riley WA, Grobbee DE (2003). Carotid intima-media thickness measurements in intervention studies: design options, progression rates, and sample size considerations: a point of view. Stroke.

[47] Amarenco P, Labreuche J, Lavallee P, Touboul PJ (2004). Statins in stroke prevention and carotid atherosclerosis: systematic review and up-to-date meta-analysis. Stroke.

[48] US Food and Drug Administration. Clinical trial imaging endpoint process standards guidance for industry. http://www.fda.gov/downloads/drugs/guidancecomplianceregulatoryinformation/guidances/ucm268555.pdf. Accessed 15 Aug 2016.

[49] Wagenknecht LE, Zaccaro D, Espeland MA, Karter AJ, O’Leary DH, Haffner SM (2003). Diabetes and progression of carotid atherosclerosis: the insulin resistance atherosclerosis study. Arterioscler Thromb Vasc Biol.

[50] Langenfeld MR, Forst T, Hohberg C, Kann P, Lubben G, Konrad T, Fullert SD, Sachara C, Pfutzner A (2005). Pioglitazone decreases carotid intima-media thickness independently of glycemic control in patients with type 2 diabetes mellitus: results from a controlled randomized study. Circulation.

[51] Dormandy JA, Charbonnel B, Eckland DJ, Erdmann E, Massi-Benedetti M, Moules IK, Skene AM, Tan MH, Lefebvre PJ, Murray GD (2005). Secondary prevention of macrovascular events in patients with type 2 diabetes in the PROactive Study (PROspective pioglitAzone Clinical Trial In macroVascular Events): a randomised controlled trial. Lancet.

[52] Hanefeld M, Chiasson JL, Koehler C, Henkel E, Schaper F, Temelkova-Kurktschiev T (2004). Acarbose slows progression of intima-media thickness of the carotid arteries in subjects with impaired glucose tolerance. Stroke.

[53] Chiasson JL, Josse RG, Gomis R, Hanefeld M, Karasik A, Laakso M (2003). STOP-NIDDM Trial Research Group: acarbose treatment and the risk of cardiovascular disease and hypertension in patients with impaired glucose tolerance: the STOP-NIDDM trial. JAMA.

[54] Green JB, Bethel MA, Armstrong PW, Buse JB, Engel SS, Garg J, Josse R, Kaufman KD, Koglin J, Korn S (2015). Effect of sitagliptin on cardiovascular outcomes in type 2 diabetes. N Engl J Med.

[55] Scirica BM, Bhatt DL, Braunwald E, Steg PG, Davidson J, Hirshberg B, Ohman P, Frederich R, Wiviott SD, Hoffman EB (2013). Saxagliptin and cardiovascular outcomes in patients with type 2 diabetes mellitus. N Engl J Med.

[56] White WB, Cannon CP, Heller SR, Nissen SE, Bergenstal RM, Bakris GL, Perez AT, Fleck PR, Mehta CR, Kupfer S (2013). Alogliptin after acute coronary syndrome in patients with type 2 diabetes. N Engl J Med.

[57] Ghosh RK, Bandyopadhyay D, Hajra A, Biswas M, Gupta A (2016). Cardiovascular outcomes of sodium–glucose cotransporter 2 inhibitors: a comprehensive review of clinical and preclinical studies. Int J Cardiol.

[58] Neal B, Perkovic V, de Zeeuw D, Mahaffey KW, Fulcher G, Stein P, Desai M, Shaw W, Jiang J, Vercruysse F (2013). Rationale, design, and baseline characteristics of the canagliflozin cardiovascular assessment study (CANVAS)—a randomized placebo-controlled trial. Am Heart J.

[59] ClinicalTrials.gov Identifier: NCT01730534. https://clinicaltrials.gov/ct2/show/NCT01730534?term=TIMI+declare&rank=1. Accessed 15 Aug 2016.

[60] ClinicalTrials.gov Identifier: NCT01986881. https://clinicaltrials.gov/ct2/show/NCT01986881?term=Ertugliflozin&rank=12. Accessed 15 Aug 2016.

[61] Wu JH, Foote C, Blomster J, Toyama T, Perkovic V, Sundstrom J, Neal B (2016). Effects of sodium–glucose cotransporter-2 inhibitors on cardiovascular events, death, and major safety outcomes in adults with type 2 diabetes: a systematic review and meta-analysis. Lancet Diabetes Endocrinol.

[62] Sattar N, McLaren J, Kristensen SL, Preiss D, McMurray JJ (2016). SGLT2 inhibition and cardiovascular events: why did EMPA-REG outcomes surprise and what were the likely mechanisms?. Diabetologia.

[63] Abdul-Ghani M, Del Prato S, Chilton R, DeFronzo RA (2016). SGLT2 inhibitors and cardiovascular risk: lessons learned from the EMPA-REG OUTCOME study. Diabetes Care.

[64] Rajasekeran H, Lytvyn Y, Cherney DZ (2016). Sodium–glucose cotransporter 2 inhibition and cardiovascular risk reduction in patients with type 2 diabetes: the emerging role of natriuresis. Kidney Int.

[65] Tahara A, Kurosaki E, Yokono M, Yamajuku D, Kihara R, Hayashizaki Y, Takasu T, Imamura M, Li Q, Tomiyama H (2013). Effects of SGLT2 selective inhibitor ipragliflozin on hyperglycemia, hyperlipidemia, hepatic steatosis, oxidative stress, inflammation, and obesity in type 2 diabetic mice. Eur J Pharmacol.

[66] Tahara A, Kurosaki E, Yokono M, Yamajuku D, Kihara R, Hayashizaki Y, Takasu T, Imamura M, Li Q, Tomiyama H (2014). Effects of sodium–glucose cotransporter 2 selective inhibitor ipragliflozin on hyperglycaemia, oxidative stress, inflammation and liver injury in streptozotocin-induced type 1 diabetic rats. J Pharm Pharmacol.

[67] Osorio H, Coronel I, Arellano A, Pacheco U, Bautista R, Franco M, Escalante B (2012). Sodium–glucose cotransporter inhibition prevents oxidative stress in the kidney of diabetic rats. Oxid Med Cell Longev.

[68] Terasaki M, Hiromura M, Mori Y, Kohashi K, Nagashima M, Kushima H, Watanabe T, Hirano T (2015). Amelioration of hyperglycemia with a sodium–glucose cotransporter 2 inhibitor prevents macrophage-driven atherosclerosis through macrophage foam cell formation suppression in type 1 and type 2 diabetic mice. PLoS One.

[69] Lin B, Koibuchi N, Hasegawa Y, Sueta D, Toyama K, Uekawa K, Ma M, Nakagawa T, Kusaka H, Kim-Mitsuyama S (2014). Glycemic control with empagliflozin, a novel selective SGLT2 inhibitor, ameliorates cardiovascular injury and cognitive dysfunction in obese and type 2 diabetic mice. Cardiovasc Diabetol.

[70] Cherney DZ, Perkins BA, Soleymanlou N, Har R, Fagan N, Johansen OE, Woerle HJ, von Eynatten M, Broedl UC (2014). The effect of empagliflozin on arterial stiffness and heart rate variability in subjects with uncomplicated type 1 diabetes mellitus. Cardiovasc Diabetol.

[71] Chilton R, Tikkanen I, Cannon CP, Crowe S, Woerle HJ, Broedl UC, Johansen OE (2015). Effects of empagliflozin on blood pressure and markers of arterial stiffness and vascular resistance in patients with type 2 diabetes. Diabetes Obes Metab.

[72] Tahara A, Takasu T, Yokono M, Imamura M, Kurosaki E (2016). Characterization and comparison of sodium–glucose cotransporter 2 inhibitors in pharmacokinetics, pharmacodynamics, and pharmacologic effects. J Pharmacol Sci.

[73] Kashiwagi A, Kazuta K, Yoshida S, Nagase I (2014). Randomized, placebo-controlled, double-blind glycemic control trial of novel sodium-dependent glucose cotransporter 2 inhibitor ipragliflozin in Japanese patients with type 2 diabetes mellitus. J Diabetes Investig.

[74] Kashiwagi A, Takahashi H, Ishikawa H, Yoshida S, Kazuta K, Utsuno A, Ueyama E (2015). A randomized, double-blind, placebo-controlled study on long-term efficacy and safety of ipragliflozin treatment in patients with type 2 diabetes mellitus and renal impairment: results of the long-term ASP1941 safety evaluation in patients with type 2 diabetes with renal impairment (LANTERN) study. Diabetes Obes Metab.

[75] Kashiwagi A, Shiga T, Akiyama N, Kazuta K, Utsuno A, Yoshida S, Ueyama E (2016). Efficacy and safety of ipragliflozin as an add-on to pioglitazone in Japanese patients with inadequately controlled type 2 diabetes: a randomized, double-blind, placebo-controlled study (the SPOTLIGHT study). Diabetol Int.

[76] Iizuka T, Iemitsu K, Takihata M, Takai M, Nakajima S, Minami N, Umezawa S, Kanamori A, Takeda H, Kawata T (2016). Efficacy and safety of ipragliflozin in japanese patients with type 2 diabetes: interim outcome of the ASSIGN-K study. J Clin Med Res.

[77] Yamamoto C, Miyoshi H, Ono K, Sugawara H, Kameda R, Ichiyama M, Yamamoto K, Nomoto H, Nakamura A, Atsumi T (2016). Ipragliflozin effectively reduced visceral fat in Japanese patients with type 2 diabetes under adequate diet therapy. Endocr J.

[78] Takahara M, Shiraiwa T, Matsuoka TA, Katakami N, Shimomura I (2015). Ameliorated pancreatic beta cell dysfunction in type 2 diabetic patients treated with a sodium–glucose cotransporter 2 inhibitor ipragliflozin. Endocr J.

[79] Ferrannini E, Muscelli E, Frascerra S, Baldi S, Mari A, Heise T, Broedl UC, Woerle HJ (2014). Metabolic response to sodium–glucose cotransporter 2 inhibition in type 2 diabetic patients. J Clin Investig.

[80] Merovci A, Solis-Herrera C, Daniele G, Eldor R, Fiorentino TV, Tripathy D, Xiong J, Perez Z, Norton L, Abdul-Ghani MA (2014). Dapagliflozin improves muscle insulin sensitivity but enhances endogenous glucose production. J Clin Investig.

[81] Olefsky JM, Kolterman OG (1981). Mechanisms of insulin resistance in obesity and noninsulin-dependent (type II) diabetes. Am J Med.

[82] Bugianesi E, McCullough AJ, Marchesini G (2005). Insulin resistance: a metabolic pathway to chronic liver disease. Hepatology.

[83] Chitturi S, Abeygunasekera S, Farrell GC, Holmes-Walker J, Hui JM, Fung C, Karim R, Lin R, Samarasinghe D, Liddle C (2002). NASH and insulin resistance: insulin hypersecretion and specific association with the insulin resistance syndrome. Hepatology.

[84] Targher G, Byrne CD (2013). Clinical review: nonalcoholic fatty liver disease: a novel cardiometabolic risk factor for type 2 diabetes and its complications. J Clin Endocrinol Metab.

[85] Nishimura N, Kitade M, Noguchi R, Namisaki T, Moriya K, Takeda K, Okura Y, Aihara Y, Douhara A, Kawaratani H (2016). Ipragliflozin, a sodium–glucose cotransporter 2 inhibitor, ameliorates the development of liver fibrosis in diabetic Otsuka Long-Evans Tokushima fatty rats. J Gastroenterol..

[86] Komiya C, Tsuchiya K, Shiba K, Miyachi Y, Furuke S, Shimazu N, Yamaguchi S, Kanno K, Ogawa Y (2016). Ipragliflozin improves hepatic steatosis in obese mice and liver dysfunction in type 2 diabetic patients irrespective of body weight reduction. PLoS One.

[87] Macha S, Mattheus M, Halabi A, Pinnetti S, Woerle HJ, Broedl UC (2014). Pharmacokinetics, pharmacodynamics and safety of empagliflozin, a sodium glucose cotransporter 2 (SGLT2) inhibitor, in subjects with renal impairment. Diabetes Obes Metab.

[88] Yale JF, Bakris G, Cariou B, Yue D, David-Neto E, Xi L, Figueroa K, Wajs E, Usiskin K, Meininger G (2013). Efficacy and safety of canagliflozin in subjects with type 2 diabetes and chronic kidney disease. Diabetes Obes Metab.

[89] Kohan DE, Fioretto P, Tang W, List JF (2014). Long-term study of patients with type 2 diabetes and moderate renal impairment shows that dapagliflozin reduces weight and blood pressure but does not improve glycemic control. Kidney Int.

